# Short‐lived peaks of stem methane emissions from mature black alder (*Alnus glutinosa* (L.) Gaertn.) – Irrelevant for ecosystem methane budgets?

**DOI:** 10.1002/pei3.10037

**Published:** 2020-12-23

**Authors:** Daniel Köhn, Anke Günther, Ines Schwabe, Gerald Jurasinski

**Affiliations:** ^1^ Landscape Ecology University of Rostock Rostock Germany

**Keywords:** Alnus glutinosa, CH_4_ budgets, methane (CH_4_), peatland, stem fluxes

## Abstract

Tree stems can be a source of the greenhouse gas methane (CH_4_). However, assessments of the global importance of stem CH_4_ emissions are complicated by a lack of research and high variability between individual ecosystems. Here, we determined the contribution of emissions from stems of mature black alder (*Alnus glutinosa* (L.) Gaertn.) to overall CH_4_ exchange in two temperate peatlands. We measured emissions from stems and soils using closed chambers in a drained and an undrained alder forest over 2 years. Furthermore, we studied the importance of alder leaves as substrate for methanogenesis in an incubation experiment. Stem CH_4_ emissions were short‐lived and occurred only during times of inundation at the undrained site. The drained site did not show stem emissions and the soil acted as a small CH_4_ sink. The contribution of stem emissions to the overall CH_4_ budget was below 0.3% in both sites. Our results show that mature black alder can be an intermittent source of CH_4_ to the atmosphere. However, the low share of stem CH_4_ emissions in both investigated stands indicates that this pathway may be of minor relative importance in temperate peatlands, yet strongly depend on the hydrologic regime.

## Graphical abstract

Stem and soil CH_4_ emissions were monitored for two years in a drained (AD) and rewetted (AW) mature alder forest. The rewetted site showed high but short‐lived stem and soil CH_4_ emissions while the drained site was neutral with respect to CH_4_ emissions. Both stem and soil emissions showed high temporal variability and maximum fluxes were at the higher end of values previously reported for temperate settings. Soil emissions always occurred during periods of repeated inundation while stem emissions only occurred once during the entire monitoring period. Overall, soil CH_4_ emissions had a much higher magnitude than stem emissions which led to a generallylow contribution of stem emissions to the total ecosystem CH_4_ budget.

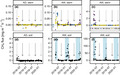

**What hypotheses or questions does this work address?**

- CH_4_ fluxes through alder stems are higher in an undrained than in a drained forested peatland.
- The relative importance of stem CH_4_ fluxes is higher at a drained peatland site because of lower soil‐borne emissions.
- Phenological events such as leaf fall and leaf out significantly increase stem CH4 emissions because they provide substrate and, thus, increase CH_4_ production in the soil whereas inundation increases the rate of internal transport inside the tree due to higher transpiration.

**How does this work advance our current understanding of plant science?**

This study advances our understanding regarding the impact of the management of undrained or dry peatland ecosystems on the magnitude and relative importance of stem‐mediated CH_4_ emissions from trees. Furthermore, it sheds light on the potential impacts of specific events such as inundation, leaf fall, leaf out, and drought on stem CH_4_ emissions.

**Why is this work so important and timely?**

Assessments of the relative importance of multi‐year stem CH_4_ emissions in undrained and dry peatland ecosystems, especially for Alder stands, are very scarce. However, the relative share of stem emissions to the CH_4_ emissions of the whole ecosystem was minor for the studied plots independent of the different upscaling and budgeting approaches tested. This contradicts previous findings attributing high importance to stem CH_4_ emissions in other ecosystems suggesting that we need much more field studies to eventually come to robust average stem contribution estimates.

## 1 INTRODUCTION

Tree stems are increasingly recognized as potential surfaces for the exchange of greenhouse gases (GHG) such as methane (CH_4_) (Pangala et al., 2013, 2015). CH_4_ is a potent GHG but its globally relevant emission sources are not yet fully understood (Melton et al., 2013; Saunois et al., 2016). Tree stems could be an important CH_4_ source in terrestrial ecosystems and may have been previously overlooked in global CH_4_ inventories (Pangala et al., 2017). Accordingly, emissions from trees have been discussed as a “new frontier in the global carbon cycle” (Barba et al., 2018).

Regionally, tree stem emissions can be the most important CH_4_ source (e.g. in the amazon basin, Pangala et al., 2017). However, the magnitude of CH_4_ fluxes from tree stems seems to vary considerably on a global scale, with fluxes tending to be lower in temperate (Gauci et al., 2010; Pitz et al., 2018) than in tropical regions (Pangala et al., 2017). The ecosystem contribution of stem CH_4_ emissions has only rarely been addressed, with the few available studies reporting contributions between 27% in a temperate and 87% in a tropical wetland (Pangala et al., 2013, 2015).

Although CH_4_ emissions from tree stems could be an emission pathway of global relevance, little information is available on the influence of ecosystem type or of abiotic factors, for example, water level and soil temperature. Also, the variability of stem CH_4_ emissions between individual trees or along the stem of the tree has rarely been studied and still needs to be studied more thoroughly (Barba et al., 2018).

Abiotic and biotic factors can influence both CH_4_ production itself and its emission rate from tree stems. Soil temperature, soil water content or water level and physiological activity of the trees are assumed to be the most important drivers of CH_4_ production (Barba et al., 2019; Schindler et al., 2020; Terazawa et al., 2015). Accordingly, a considerable increase in stem CH_4_ emissions has been associated with increasing water tables (Pitz et al., 2018; Schindler et al., 2020) and increasing soil and air temperatures (Barba et al., 2019). This is further backed by mesocosm experiments (Pangala et al., 2014; Rusch & Rennenberg, 1998). In addition, canopy conductance and photosynthetic photon flux density can be closely related with CH_4_ emissions measured above the forest canopy, pointing towards an influence of tree physiological activity (Deshmukh et al., 2020; Tang et al., 2018).

The CH_4_ emitted through tree stems originates from either the tree trunk itself or the soil. Several studies found stems of upland trees to be sources of CH_4_ despite rooting in very dry soils (Machacova et al., 2016; Maier et al., 2017; Pitz & Megonigal, 2017; Wang et al., 2016) suggesting production within the tree. In such upland settings, heartwood rot has been shown to be a good indicator for stem CH_4_ emissions (Covey et al., 2012; Wang et al., 2016). In addition, methanogenesis may also take place in aerobic parts of plant tissue (Bartlett et al., 2006), and newer results on alternative methane producing bacteria (Bižić et al., 2020) shows that there is still much to be learned about methanogenic pathways. CH_4_ produced in the soil can enter the tree through the roots and may be transported through the sap or through air‐filled spaces in the trunk (Schröder, 1989). The results of several studies finding higher CH_4_ emissions in the lowest parts of the trunk (Barba et al., 2019; Schindler et al., 2020; Terazawa et al., 2007) suggest that trees may often be just conduits for the transport of gas that is produced in the soil.

Wetland trees like black alder (*Alnus glutinosa* (L.) Gaertn.) additionally have the ability to transport oxygen to the rhizosphere and thereby stimulate CH_4_ consumption (Joabsson & Christensen, 2001). Furthermore, alder trees predominantly grow on peat soils in temperate regions, which themselves have a high potential for CH_4_ emissions (Turetsky et al., 2014). Hence, alder trees may constitute an important additional, so far neglected pathway of CH_4_ emissions from temperate peatlands. Black alder forests cover roughly 5% of all central European forests (Claessens et al., 2010). In north‐eastern Germany, black alder is even the spatially second most important deciduous tree species (after the European beech, *Fagus sylvatica* L.) and its relevance could increase even further in the future since it has a high potential for sustainable silviculture on rewetted peatlands (Schäfer et al., 2005). Being a widespread tree species of economic importance, the impact of mature alder forests needs to be thoroughly investigated and integrated into local and regional GHG budgets.

Here, we study patterns of CH_4_ emissions from stems of mature black alder trees and relate them to soil CH_4_ emissions to evaluate the relative contribution of CH_4_ passing through trees in two temperate alder forests over 2 years. One of the studied forests is currently drained (“alder drained”, AD) while the other is undrained (i.e., “alder wet”, AW) allowing us to study the influence of wet versus dry conditions. Furthermore, we studied the possible effects of leaf fall on soil CH_4_ emissions by determining CH_4_ production potentials in an incubation experiment. We tested the following hypotheses:

1. CH_4_ emissions from stems of black alder contribute significantly to the overall CH_4_ budget.
2. Absolute values of tree stem CH4 emissions of the undrained site (AW) are larger than at the drained site (AD), while relative contributions are higher at AD due to low or no emissions from the soil.
3. Events like inundation, leaf fall or leaf out significantly increase stem CH4 emissions, because inundation increases CH4 production, while leaf fall provides substrate for methanogenesis and leaf out increases rates of internal transport in the tree due to higher transpiration.

## 2 MATERIAL AND METHODS

### 2.1 Study sites

**FIGURE 1:**
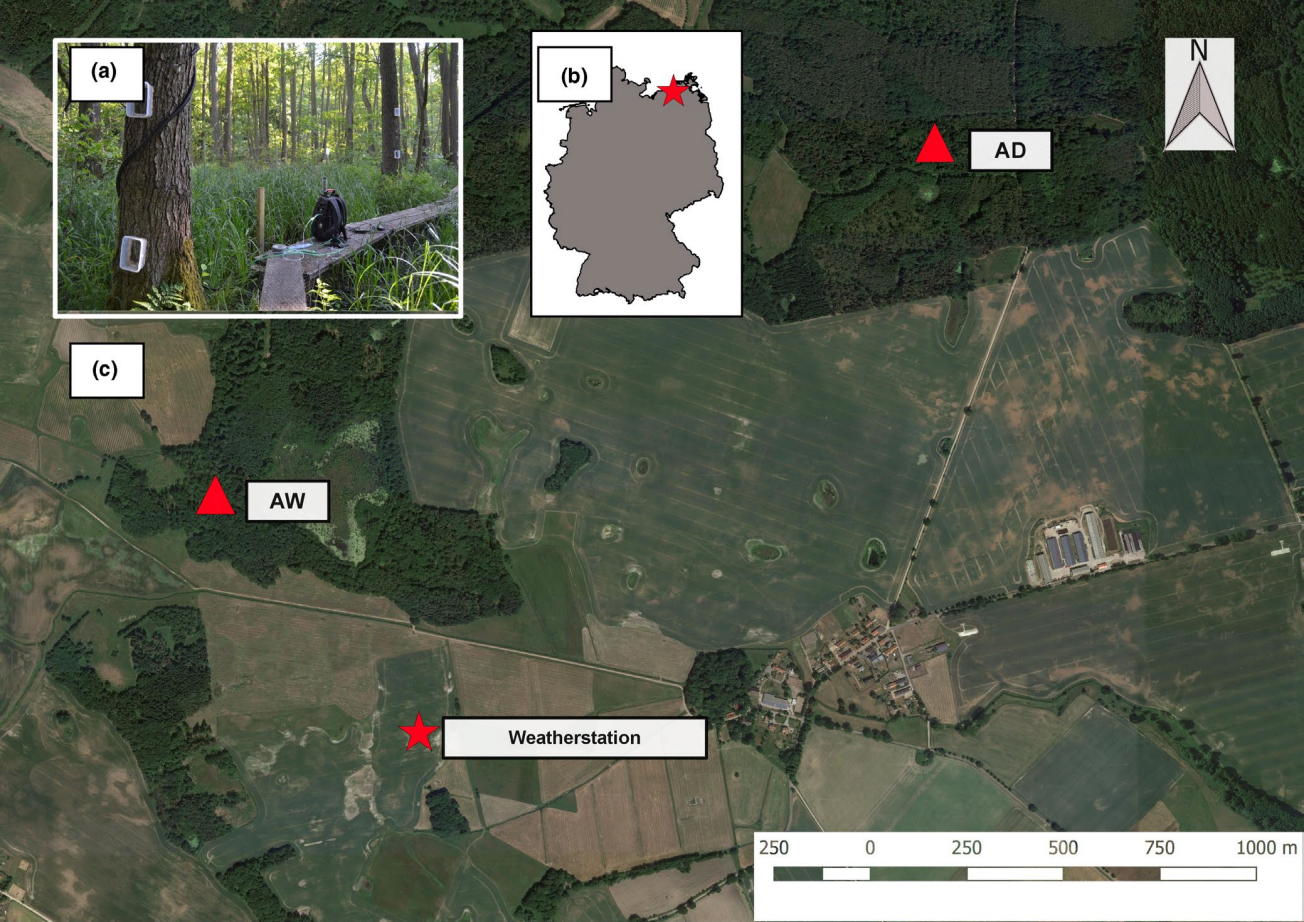
Location and setup of the wet (Alder Wet, AW) and dry (Alder Dry, AD) alder forests in north‐eastern Germany. (a) Stem chambers mounted on black alder at AW together with field CH_4_ analyzer (in background), (b) location of study sites in Germany, and (c) location of study sites AW and AD relative to weather station. Satellite imagery © Google satellite

Both studied sites are located in north‐eastern Germany (54° 7’ 36.27” N, 12° 28’ 55.5” E) sharing a distance of approx. 1 km (Figure 1). AD has been logged for over 200 years and was most likely drained even earlier. After decades of drainage AW has gradually been rewetted since the 1990s with the current water levels establishing in 2004. Both sites are located in local depressions approx. 40 m.a.s.l. receiving 627 mm of rainfall on average (derived from the 1 km grid product of the German Weather Service, Krähenmann et al. (2016), DWD, reference period: 1981–2010) and showing an annual mean temperature of 8.5°C. The research sites are fenced areas of approx. 12 × 30 m with a boardwalk running along the long side through the centre of the short side. Boardwalks were constructed between April and June 2017.

Black alder (*Alnus glutinosa* (L.) Gaertn.) is the only tree species in AW. The understorey features sedges (*Carex acutiformis* Ehrh., *C. riparia* Curtis), featherfoil (*Hottonia palustris* L.) and bitter nightshade (*Solanum dulcamara* L.). Temporal flooding creates areas of open water. Peat depth is around 2 m with an average soil pH of 5.1. Mass related soil carbon and phosphorus contents are 38% and 1.4% (dry weight), respectively (Jurasinski et al., 2020).

AD features a mixed stand of black alder and European ash (*Fraxinus excelsior* L.). The understory mainly comprises stinging nettle (*Urtica dioica* L.), European elder (*Sambucus nigra* L.), and blackberry (*Rubus* sp. L.). In spring lesser calendine (*Ranunculus ficaria* (HUDS.)) is dominant on the ground. The peat is very shallow with a depth of approximately 40 cm. Soil pH is 4.5 with soil carbon and phosphorus content being 19% and 2.7% (dry weight), respectively (Jurasinski et al., 2020).

### 2.2 Study setup

We used flow‐through non‐steady‐state chambers to measure gas exchange between the atmosphere and the stem or soil surface, respectively (Livingston & Hutchinson, 1995). We measured fluxes of soil GHG exchange in both sites at five different locations with permanently installed PVC collars in the ground (approx. 10 cm depth, radius = 65 cm, height = 0.9–1.4 m, Günther et al., 2014). The chambers for the ground measurements were equipped with three fans as well as a temperature and humidity sensor. Open‐top stem chambers were permanently installed on three trees at three heights (approx. 0.3 m, 1 m, 2 m) above the stem base at each site (i.e., 9 chambers per site, Figure 1). We constructed the stem chambers from polypropylene containers (EMSA, Emsdetten, Germany, V = 0.001 m^3^) by cutting out the bottom and mounting the chambers onto the tree stems with an adhesive synthetic sealant (plastic fermit, Fermit GmbH, Vettelschoß, Germany).

### 2.3 Flux measurements

Measurements were carried out every 2 weeks over the course of 2 years between 3 May 2018 and 30 April 2020. On 26 June 2018 we ran an 18‐hr campaign to determine differences in CH_4_ exchange from tree stems during day and night. During this campaign, all stem chambers were measured every 45 min.

For measurements of soil GHG exchange, the flexible side walls of the chamber were attached to the PVC collar with a rubber band. For stem chamber measurements, the chamber was closed using a lid with a rubber gasket. Inlet and outlet tubes (PVC, inner diameter: 3.4 mm) connected the chambers to a portable gas analyser. Carbon dioxide (CO_2_) and CH_4_ concentrations inside the chamber were measured by laser spectrometers (“Ultra‐Portable Greenhouse Gas Analyzer”, Los Gatos Research, Mountain View, USA and “GasScouter”, Picarro, Santa Clara, USA) with a measurement frequency of 1 Hz. Enclosure time for soil and stem chambers was 5 and 3 min, respectively. A shorter enclosure time for the stem chambers was chosen due to the much smaller headspace volume (0.002 m^3^ vs. 0.31 m^3^). Flux estimation was performed using R 3.6.4 (R Development Core Team 2020) and an updated version of the *fluxx*() function in the package *flux* (Jurasinski et al., 2014). Slopes were calculated between all concentration measurements during chamber closure. Subsequently the median of the slope values was used for the further calculation steps of the flux estimation (i.e., median based regression; Siegel, 1982).

### 2.4 Additional measurements

A weather station (CR300, Campbell Scientific GmbH, Bremen, Germany) located 500 m southeast of AW (see Figure 1) recorded air temperature, wind speed, precipitation and photosynthetic photon flux density (PPFD). At both sites temperature loggers (‘HOBO Pendant’, Onset, Bourne, USA), installed at three different locations at 5 cm and 15 cm depth, recorded soil temperature every 15 min. Water levels were automatically logged (‘CS457 dipper PT’, SEBA Hydrometrie GmbH & Co. KG, Kaufbeuren, Germany) at 1‐min intervals at AW and AD. Accounting for differences in expected water level fluctuations between the sites, loggers were installed at different depths so that water levels could be detected in the ranges from ~50 cm above the soil surface to 70 cm and 280 cm below soil surface for AW and AD, respectively.

### 2.5 Methane budgets

Since we were not able to model annual CH_4_ budgets using ancillary data, we based budgeting on a statistical approach adapted from Günther et al., (2017).We used the area‐under‐curve function (*auc*.*mc*) from the R package *flux* (Jurasinski et al., 2014) to integrate flux values over time by linear interpolation. For each measurement day, one flux value was randomly chosen per flux subset (stem or soil) and site (AD or AW). This was repeated 100 times to obtain 100 flux time series. Then, the area‐under‐curve was calculated 100 times for each flux time series, each time leaving out one flux value (jackknife method), leading to a total of 10,000 annual CH_4_ balances. For the final CH_4_ balances we calculated the average and standard deviation of all 10,000 balances.

For determining the contribution of stem and soil CH_4_ emissions, we projected the average stem emissions (*F_stem,_* Equation 3) of the lower 2.2 m of the stem onto the base area of the respective tree trunks. The upper limit of 2.2 m was chosen because we assumed that no CH_4_ was emitted from the tree trunks above that height. Based on findings of related studies on tree stem emissions (Pangala et al., 2013, 2017; Schindler et al., 2020) and our own data showing a strong decrease of CH_4_ emissions with increasing height (see Results), we assume the error introduced by this simplification to be negligible. First, we measured the stem diameters of all sampled trees at the stem base and at 2.2 m height. Then, we calculated the surface area of the lower 2.2 m of each tree (A_Surface_) by using Equation (1) that assumes the tree trunk to be a circular conical frustum:

(1) $$A_{\text{Surface}}=\left(r_{\text{Base}}+r_{\text{Top}}\right)\times \pi \times \sqrt{{\left(r_{\text{Base}}‐r_{\text{Top}}\right)}^{2}+h^{2}}$$

where r_Base_ and r_Top_ are the base and top radii (m) and *h* is the height (2.2.m). The base area of each tree trunk was calculated by Equation (2):

(2) $$A_{\text{Base}}=\pi \times {\left(r_{\text{Base}}\right)}^{2}$$

Finally, we calculated the stem CH_4_ flux projected to ground surface (F_Base_) using Equation (3)

(3) $$F_{\text{Base}}=\frac{F_{\text{Stem}}\times A_{\text{Surface}}}{A_{\text{Base}}}$$

where F_stem_ is the average flux estimated from stem chambers relative to the stem area. Because no consensus exists on how to upscale stem fluxes to the ecosystem scale, we compared three different upscaling approaches for calculating stem emissions per hectare:

A1. We used F_Base_ to calculate annual balances per site and then upscaled by multiplying with the approximate total base area of all trees per hectare.

A2. We used the daily average stem flux of each tree and multiplied it with the respective surface area of the stem to get the average flux per tree and day. With this we calculated combined annual balances for the three sampled trees of each site, and then multiplied with number of trees per hectare.

A3. We used the overall average of stem fluxes over the measurement year and multiplied it with the total approximate stem surface area of all tree stems in one hectare. Then, we projected the per‐hour flux to the period of one entire year by simple multiplication (8 760 hr in a year).

For each approach we calculated the contribution of stem emissions with respect to the total emissions of the ecosystem (stem and soil combined). For calculating the contributions, we used the calculated balances for soil CH_4_ emissions (A1 and A2) or the overall average soil CH_4_ flux (A3). Soil CH_4_ fluxes and soil CH_4_ balances were spatially upscaled by multiplying with 1 ha excluding the base area of all trees per ha (AD: 273 trees ha^‐1^ with 137 m^2^ base area, AW: 311 trees ha^‐1^ with 106 m^2^ base area).

### 2.6 Incubation experiment

We complemented our field monitoring data with an incubation experiment, to study the possible effects of leaf fall on soil CH_4_ emissions. Soil samples were taken at both sites from the top soil (0–10 cm) in January 2020 and stored at approx. 5°C in a fridge for 9 days. Alder leaves were collected in fall 2018, dried for 3 days at 60°C and subsequently stored in air‐tight plastic bags. Leaves were then ground to 0.5 mm particle size in order to be able to add the same amount of leaves and provide a homogenous mixture in every incubation container. The incubation experiment comprised three different anaerobic incubation treatments to determine the CH_4_ production potential of soil and leaves separately and in combination: Each incubation container (140 ml, Weck, Wehr‐Öffllingen, Germany) received either 10 g soil (Soil_only_), 50 mg leaves (Leaves_only_), or a combination of 10 g soil and 50 mg leaves (Soil* + *Leaves_comb_). To establish anaerobic conditions 10 ml of de‐ionized water were added to the containers, after which the containers were closed with a gas‐tight lid. Then, the headspace was purged for 30 s with pure nitrogen (N_2_) gas. The containers remained closed during the entire experiment.

In total, the experiment ran for 34 days. Gas samples were taken at two‐day intervals at the beginning and later at four‐ to six‐day intervals. Gas samples (approx. 60 ml) were taken from the containers with a 60 ml syringe. Approximately 25 ml headspace gas was added to pre‐evacuated 12 ml glass vials (Exetainer, Labco Ltd., Lampeter, UK). After each sampling the incubation containers were purged with N_2_ gas for 30 s. The analysis of the gas samples was carried out with a gas chromatograph (GC 2010, Shimadzu, Kyoto, Japan) within three days after sampling.

We combined the CH_4_ production potentials of the incubations of Soil_only_ and Leaves_only_ when comparing with Soil* + *Leaves_comb_ in order to investigate the effect of the addition of leaves on the CH_4_ production potential in comparison to the production potential of the materials on their own. All production potentials were calculated in relation to the initial mass of the incubated material.

The production potential of the soil and leaves only incubation (P_p_‐Soil_only+_Leaves_only_) was calculated as follows:

(4) $$P_{p}‐{\text{soil}}_{\text{only}}+{\text{Leaves}}_{\text{only}}=\frac{\left(C_{S}‐C_{0}\times V_{H}\times M_{CH_{4}}\right)/t_{i}}{V_{M}}\times m_{p}+\frac{\left(C_{L}‐C_{0}\times V_{H}\times M_{CH_{4}}\right)/t_{i}}{V_{M}}\times m_{p}$$

And the production potential of the combined incubation of soil and leaves (P_p_‐Soil* + *Leaves_comb_) as follows:

(5) $$P_{p}‐\text{Soil}+{\text{Leaves}}_{\text{combination}}=\frac{\left(C_{S+L}‐C_{0}\times V_{H}\times M_{CH_{4}}\right)/t_{i}}{V_{M}}\times m_{p}$$

where *C_S_* and *C_L_* are the CH_4_ concentrations in the headspace samples of the incubated soil and leaves, respectively, and *C_S+L_* is the CH_4_ concentration in the headspace of the incubated combination of soil and leaves in combination. *C_0_* is the starting concentration of CH_4_ (assumed to be 0), *V_H_* is the headspace volume (approx. 120 ml), *V_M_* is the molar volume of CH_4_ (24.7 L mole^‐1^ at 25°C), *M_CH4_* is the molar mass of CH_4_ (16.04 g mole^‐1^), *t_i_* is the duration of the incubation, and *m_p_* is the mass of the incubated material.

### 2.7 Statistical analyses

Statistical analyses and visualizations were carried out with R 3.6.4 (R Development Core Team 2020). The entire datasets and subsets were tested for normality using the Shapiro Wilk test. We used Kruskal–Wallis’ test to test for differences between groups of the non‐normally distributed data. All tests were run against an alpha level of 0.05. Simple linear and exponential linear relationships between abiotic variables (soil temperature, air temperature, and water level) with stem and soil CH_4_ fluxes were investigated. The performance of the models was compared based on R^2^. Additionally, subsets of stem fluxes with R^2^ > 0.75 were used to evaluate relationships between stem CO_2_ fluxes (R^2^ > 0.75) and height above stem base (HASB). The coefficient of determination larger than 0.75 was chosen because we thereby excluded all measurements where there was no flux apparent which may have distorted a potentially functional relationship between stem CO_2_ flux and HASB with stem CH_4_ flux.

## 3 RESULTS

### 3.1 Environmental conditions

**FIGURE 2:**
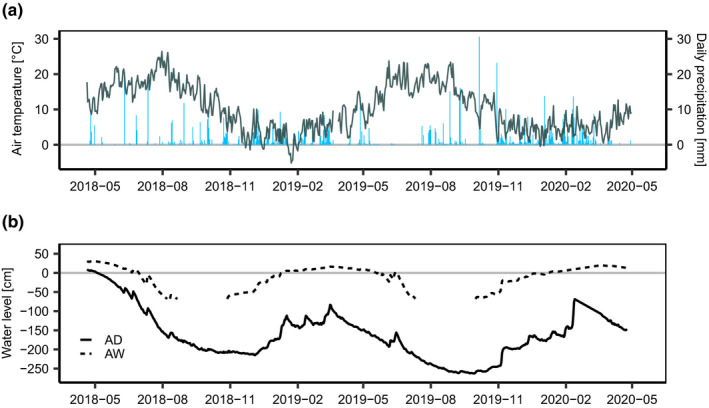
Seasonal course of (a) daily mean air temperature and daily precipitation, and (b) water level at the wet (Alder Wet, AW) and dry (Alder Dry, AD) alder forest. The solid line represents water level at AD, the dashed line water level at AW. Note: Missing values represent periods where water level fell below the level at which the gauge was installed

The study period was characterized by a large precipitation deficit together with above average temperatures compared to the respective average values of the climate period 1981–2010 (derived from the 1 km grid product of the German Weather Service, Krähenmann et al. (2016), DWD, reference period: 1981–2010). In the first study year (May 2018–April 2019) the total precipitation was 381 mm (60% of long‐term average) and 424 mm (67% of long‐term average) in the second year (May 2019–April 2020, Figure 2). In both years, the deficit was especially prevalent in spring and summer. The average air temperature was 10.4°C and 10.9°C for year one and two (1.7°C and 2.2°C above the long‐term annual mean temperature). The water table was very variable at both the AD and AW. Despite being inundated repeatedly in winter, AW showed water levels up to approximately −70 cm. Shortly before monitoring started AD had water levels above the soil surface, however, the site was deeply drained during most of the monitoring period with water levels down to −270 cm (Figure 2).

### 3.2 Flux measurements

**FIGURE 3:**
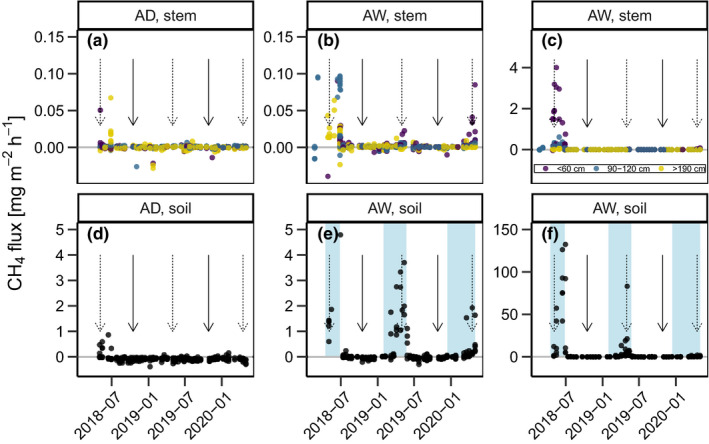
Seasonal course of (a)–(c) stem CH_4_ emissions from three different height categories and (d)–(f) soil CH_4_ emissions at the drained (Alder Dry, AD) and undrained (Alder Wet, AW) alder forests. Panels (c) and (f) show the entire range of CH_4_ fluxes in AW, while panels (b) and (e) are zoomed in on small fluxes (0–0.1 mg^‐1^m^‐2^hr^‐1^). Blue shading in panels (e) and (f) indicate inundation. Vertical dashed arrows indicate leaf‐out, solid arrows depict leaf fall by approximate date. Please note the differing scales on the y‐axes

In total, we measured 1,167 stem flux and 414 soil fluxes. Soil as well as stem CH_4_ fluxes were significantly higher in AW than in AD (*p* < .01, χ^2^ = 140.83, *df*: 1 and *p* < .01, χ^2^ = 119.26, *df*: 1, respectively). During the study period, AD tended to show a small CH_4_ uptake by the soil with an average flux of −0.1 ± 0.1 mg^‐1^m^‐2^hr^‐1^ (all values reported with ±*SD*), while CH_4_ exchange was not detectable at the tree stems. Meanwhile, CH_4_ emissions were temporarily high both from the soil and the stem surface in AW (4.8 ± 18.8 mg^‐1^m^‐2^hr^‐1^ and 0.1 ± 0.3 mg^‐1^m^‐2^hr^‐1^, respectively). However, emissions from both stems and soil in AW were very short‐lived and mostly lasted for only 6–8 weeks a year without showing further seasonal patterns. The highest stem CH_4_ flux was 4.0 mg^‐1^m^‐2^hr^‐1^ (relative to stem area) on 15 May 2018 while the highest recorded soil CH_4_ flux was 132.4 mg^‐1^m^‐2^hr^‐1^ on 29 June 2018 (relative to ground area, Figure 3). An intensive measuring campaign over 18 hr of high‐frequency measurements revealed no diurnal pattern of CH_4_ emissions from the tree stems at AW (see Figure S3). Overall, CH_4_ emissions from the individual trees sampled in AW differed significantly from each other (*p* < .01, χ^2^ = 6.635 *df*: 2). Stem CH_4_ emissions were significantly lower higher up the stem (*p* < .01, χ^2^ = 16.873, *df*: 2, Figure 4).

**FIGURE 4:**
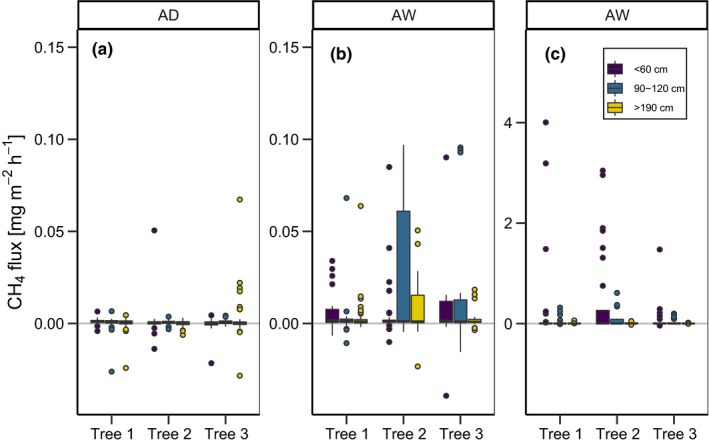
Stem CH_4_ fluxes by site and height group at (a) AD (Alder Dry) and (b) and (c) at AW (Alder Wet). Panel (c) shows the entire range of the stem CH_4_ fluxes at AW, while panel (b) shows only small fluxes in the range 0–0.1 mg^‐1^m^‐2^hr^‐1^

Stem CH_4_ emissions varied significantly with diameter at breast height in both AD and AW (*p* < .01, χ^2^ = 16.59, *df*: 2 and *p* = .03, χ^2^ = 6.64, *df*: 2). During times when stem CH_4_ emissions could be detected (fluxes with R^2^ > 0.75), a weak linear relationship between the height above the stem base (HASB) and stem emissions could be observed (R^2^ = 0.01, *p* < .1, *df*: 63, also see Figure S2, Table S2). Due to the erratic patterns of the stem and soil emissions, relationships between soil temperature or air temperature were generally weak. Water level, soil and air temperature all had a weak but significant influence on stem CH_4_ emissions. An exponential relationship between air temperature and stem CH_4_ emissions had the best explanatory power (R^2^ = 0.24, *p* < .01, Table S3). Soil CH_4_ emissions were most strongly exponentially correlated with soil temperature (R = 0.26, *p* < .01), however, also air temperature and water level showed weak but significant relationships (Table S3). Soil CH_4_ emissions tended to be highest when the water level was just above the soil surface, while stem CH_4_ emissions were higher when the soil was inundated by approximately 25 cm (Figures 2 and 3).

### 3.3 CH_4_ budgets

Annual CH_4_ budgets differed strongly between stem and soil and between measuring years at AW. During year one the soil CH_4_ budget for AW was higher by a factor of 20 compared to the second year (Table 1).

**TABLE 1:** Absolute annual balances and ecosystem CH_4_ budget contribution of stem CH_4_ emissions^vi^ at AD and AW in year one and two

	AD					
	Year 1^a^			Year 2		
Approach^b^	soil_absolute_ ^c^	stem_absolute_ ^d^	share_stem_ ^e^	soil_absolute_	stem_absolute_	share_stem_
A1	−2.9 ± 0.7	0.002 ± 0.002	0.07	−6.8 ± 0.3	0.004 ± 0.001	0.06
A2	−2.9 ± 0.7	0.001 ± 0.002	0.03	−6.8 ± 0.3	0.004 ± 0.001	0.07
A3	−4.1 ± 5.7	0.007 ± 0.063	0.17	−7.8 ± 13.2	0.005 ± 0.020	0.05

	AW					
	Year 1			Year 2		
Approach	soil_absolute_	stem_absolute_	share_stem_	soil_absolute_	stem_absolute_	share_stem_
A1	753 ± 93	1.2 ± 0.1	0.16	51 ± 10	0.01 ± 0.001	0.02
A2	753 ± 93	2 ± 0.2	0.26	51 ± 10	0.07 ± 0.002	0.14
A3	120 ± 358	0.17 ± 0.75	0.14	20 ± 120	0.004 ± 0.014	0.02

Note

^vi^All values are given in ± 1 *SD*.

^a^ Year 1 depicts the period 1 May 2018 to 30 April 2019, Year 2 depicts the period between 1 May 2019 and 30 April 2020.

^b^ Approaches for upscaling: A1: Stem CH_4_ emissions of the stem projected to the base area and multiplied with the number of trees per hectare. A2: Daily average of stem CH_4_ emissions multiplied with the surface area of the stem – Average flux per day and tree. A3: Overall average of stem CH_4_ fluxes multiplied with the approx. total surface area of all tree stems projected to the period of 1 year.

^c^ Total CH_4_ balance for the soil measurements [kg^‐1^ha^‐1^year^‐1^]

^d^ Total CH_4_ balance for the stem measurements [kg^‐1^ha^‐1^year^‐1^]

^e^ Relative share of stem CH_4_ emissions of total CH_4_ emissions [%]

All upscaling approaches yielded similar results regarding absolute annual balances and relative contributions of tree stems. The contributions of stem CH_4_ emissions to overall balances were very low, not exceeding 0.26% in any of the upscaling approaches. Overall, the relative contribution of stem CH_4_ emissions decreased strongly in the second study year, despite simultaneously decreasing soil CH_4_ emissions. Additionally, contributions of stem CH_4_ emissions were lower in AD than in AW, despite the overall lower soil CH_4_ emissions in AD.

### 3.4 Effect of leaves and leaf fall on methane production

**FIGURE 5:**
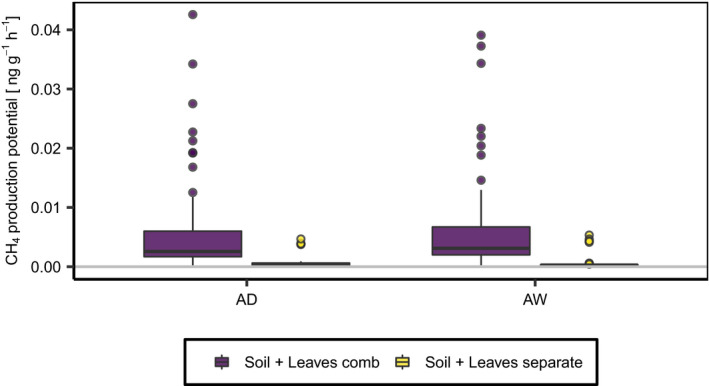
CH_4_ production potential of the different incubation treatments for the wet (AW) and dry (AD) alder forests

In the incubation experiment adding black alder leaves generally increased the CH_4_ production potential of the soil. For incubations of Soil + Leaves_comb_, CH_4_ production increased by 924% and 774% using materials from AW and AD, respectively, compared to the combined potential of Soil_only_ and Leaves_only_ (Figure 5). CH_4_ production in the containers increased following a four‐day lag phase and peaked after 14 days. Thereafter, CH_4_ production decreased gradually and ceased entirely after 30 days of incubation. The production potential in Soil + Leaves_comb_ did not differ significantly between the sites (*p* = .41, χ^2^ = 0.681, *df*: 1).

Despite the results from the incubation experiment, soil CH_4_ fluxes in the field showed no increase after leaf fall at AD or AW (Figure 3). In 2018, CH_4_ fluxes at AW were slightly higher during the four weeks after leaf fall compared to the four weeks prior to leaf fall (*p* = .03, χ^2^ = 4.505, *df*: 1). No significant difference of CH_4_ fluxes before and after leaf fall could be observed at either of the other leaf fall events (AW in 2019: *p* = .25, χ^2^ = 1.329 *df*: 1, AD in 2018: *p* = .21, χ^2^ = 1.572 *df*: 1, or in 2019: *p* = .37, χ^2^ = 0.795 *df*: 1).

## 4 DISCUSSION

### 4.1 The importance of stem CH_4_ emissions

Our results show that stems of mature black alder trees act as conduits for CH_4_ and can, at least temporarily, contribute a relevant share of overall ecosystem CH_4_ emissions. Overall, contributions of stem emissions were low compared to other studies (Pangala et al., 2013, 2015), regardless of the upscaling approach that was used. Only in AD, where soil CH_4_ exchange mainly consisted of small CH_4_ uptake, the results of the three approaches differed up to one order of magnitude. Even during peak emissions from trees in AW in spring 2018, ecosystem shares of stem CH_4_ emissions were lower than 1% due to the simultaneously very high soil emissions. 87% of all stem CH_4_ emissions in year one occurred during this two‐month period between 1 May and 1 July of 2018 (calculated using approach A1). This suggests a potentially transient nature of stem CH_4_ emissions. It is important to note that individual soil CH_4_ fluxes during spring 2018 were extremely high compared to other studies from temperate wetlands (Pangala et al., 2015; Turetsky et al., 2014). However, average CH_4_ fluxes from soil at AW in this study (4.8 mg^‐1^m^‐2^hr^‐1^) fit very well to the mean for emissions from temperate peatlands (4.5 mg^‐1^m^‐2^hr^‐1^, Turetsky et al., 2014). The weak average CH_4_ uptake of the soil at AD (−0.1 mg^‐1^m^‐2^hr^‐1^) is slightly higher than CH_4_ uptake rates in upland forests that were previously reported (−0.01 mg^‐1^m^‐2^hr^‐1^, Pitz & Megonigal, 2017).

For future studies it would be interesting to estimate the contribution of stem CH_4_ emissions in a constantly inundated alder forest where stem emissions may persist while soil emissions may be hampered by oxidation in the water column (Bastviken et al., 2002, 2008). In such environments, the importance of stem CH_4_ emissions could be much higher. In addition, the large variability of stem CH_4_ fluxes calls for more long‐term monitoring to be able to predict times of peak emission from tree stems. One interesting factor would be to study the potential lag effects of water table height, since stem and soil CH_4_ emissions in 2018 did not peak simultaneously in our study. Stem emissions were highest when the soil was inundated while soil emissions peaked when the water level had already receded to the soil surface. One reason for this could be that during inundation large proportions of the CH_4_ produced in the soil can be oxidized in the water column (Bastviken et al., 2008). During these times the emission path via uptake of the roots could represent a preferential CH_4_ emission pathway of major importance.

### 4.2 Drained versus undrained alder forest

Despite drought conditions influencing water levels in both study sites, AW on average had distinctively higher CH_4_ emissions from soil and stems than AD. Our results therefore support the high importance of water level also for stem CH_4_ emissions (Barba et al., 2019; Pitz et al., 2018; Schindler et al., 2020). Interestingly, stems at AD did not emit CH_4_ and therefore did not offset the sink function of the soil, contrary to what was found at other dry forests (Pitz & Megonigal, 2017; Wang et al., 2016). It is possible that the tree roots were not connected to the groundwater as rooting depth of black alder varies considerably (Pietzarka & Roloff, 2000), or that CH_4_ production was very low in the water‐saturated deep soil due to low carbon content. Additionally, it may be of relevance when the alder trees developed. Before drainage the roots of the alder may not have rooted as deeply and were therefore disconnected from the groundwater by drainage.

### 4.3 Influence of inundation, drought, leaf out, and leaf fall on stem CH_4_ emissions

Our data indicate that most likely a combination of inundation, increasing temperatures and increasing physiological activity of vegetation and/or microorganisms leads to higher emission rates of CH_4_ from both soil and stem surfaces. This is in line with the most important influencing factors for CH_4_ emissions from stems found in other studies (Barba et al., 2019).

It is possible that thresholds regarding the duration of inundation must be exceeded before stem CH_4_ emissions occur, as indicated by the lack of emissions from stem surfaces following a shorter period of inundation in spring 2019 compared to the peak emissions in spring 2018. Longer periods of inundation could be required to sufficiently deplete the oxygen and other less energy‐yielding alternative terminal electron acceptors like nitrate in the soil (Dean et al., 2018). Especially in Alder stands, nitrate concentrations, and, thus, the availability of alternative terminal electron acceptors, may be high because of the nitrogen fixation abilities of the Alder trees in symbiosis with bacteria of the genus *Frankia* (Beaupied et al., 1990). Also, increases in methanogen activity may start with a lag time, as shown in incubation experiments (Sun et al., 2012; Ye et al., 2015). During low concentrations and production rates of CH_4_ in the soil, transport inside the tree is likely dominated by passive diffusion (Kutschera et al., 2016). Hence, it is possible that in 2019, when fluxes and presumably soil CH_4_ concentrations were low, CH_4_ was primarily released via the soil‐atmosphere interface because of the slow diffusive transport inside the trees. Contrastingly, in 2018 the CH_4_ concentrations in the soil were likely higher, potentially triggering a more effective mode of transport inside the trees (Kutschera et al., 2016), leading to higher stem fluxes. However, this explanation is based upon the assumption that all emitted CH_4_ is produced in the soil. The remaining uncertainty regarding the exact nature of CH_4_ transport inside the tree calls for further investigations.

Climatic conditions during the study period were very variable. The summer drought conditions entirely stopped CH_4_ emissions from both stems and soils and fostered CH_4_ uptake in both sites. Conditions at AW even changed so drastically that, despite showing strong CH_4_ emissions in summer 2018, the soil turned to a sink in the following summer with a maximum CH_4_ uptake of −0.3 mg^‐1^m^‐2^hr^‐1^ in July 2019, which was similar to the uptake rate at AD. The relatively high abundance of methanotrophic bacteria at AW (Weil et al., 2020) may provide a good explanation for the observed patterns. Since global warming increases climate variability and the likelihood for extreme events like drought and strong precipitation (King & Karoly, 2017), the importance of stem CH_4_ emissions could change drastically in the future or at least could vary quite strongly across years.

The observed emission peaks took place during times of leaf out of the black alder. Thus, a rise in tree physiological activity and transpiration rate possibly caused more CH_4_ to enter the trees. Sap flow as a proxy for transpiration can explain stem CH_4_ emissions (Barba et al., 2019). Further, the results of Barba et al. (2019) suggest that increasing temperatures also directly lead to an increase in stem CH_4_ emissions. Thus, it is likely that a combination of sufficient water availability, high rates of sap flow and rising temperatures led to peak stem CH_4_ emission in our study. In addition, stem CO_2_ emissions can be an indicator for stem respiration and hence for tree physiological activity (Hölttä & Kolari, 2009; Machacova et al., 2019), explaining stem CH_4_ emissions (Barba et al., 2017). In our study, stem CO_2_ emissions could only explain 10% of all CH_4_ fluxes with R > 0.75 at AW (tested with simple linear regression, see Figure S1, Table S1). Hence, stem CH_4_ emissions in our study seem to be independent from the physiological activity of the tree. This is further supported by our finding that fluxes did not vary over the course of the day (see Figure S3), although the lack of diurnal variation could also be explained by overall small stem fluxes during the intensive sampling campaign. Thus, we cannot support the assumptions of Tang et al., (2018) and Deshmukh et al., (2020) on diurnal variability of ecosystem CH_4_ flux being determined by the variability of CH_4_ emissions from trees.

Additionally, our data reveal large individual differences in stem CH_4_ emissions between trees, despite all trees being of the same species and of similar size. Thus, other factors that were not considered in this study are likely important for stem CH_4_ emissions. For example, differences in microbiological community and soil microtopography may influence methanogenesis and, thus, pore water CH_4_ concentration in the rooting zones of the individual trees (Pangala et al., 2015; Terazawa et al., 2015). Physiological activity inside the tree (Covey et al., 2012; Zeikus & Ward, 1974) or morphological parameters such as wood density could also be responsible for different rates of CH_4_ emission between individual trees (Pangala et al., 2013).

Finally, as indicated by our incubation experiment, leaf fall holds the potential to increase CH_4_ production, probably by providing fresh substrate for methanogenesis. However, the results from the incubation are not directly transferable to the field, as fluxes measured in the field did not increase in the weeks after leaf fall. In the field, both temperature, water availability and delayed decomposition may have been limiting factors for methanogenesis despite the input of potential substrate. Hence, it is unlikely that leaf fall alone leads to CH_4_ emission peaks in temperate regions. In the incubation experiment the leaves were ground, providing easily degradable substrate in a water‐saturated, anoxic environment with temperatures of around 25°C. In contrast, water levels during leaf fall in the field were constantly below the soil surface and soil temperatures ranged around 10°C. Since temperature (e.g. Koebsch et al., 2013) and the availability of strongly decomposed organic residues (Dean et al., 2018) are known to exert a strong control on methanogenesis, the effects of leaf fall on CH_4_ emissions should be studied through combinations of leaf‐exclusion treatments and incubation/mesocosm experiments in the future. The potential for leaf fall increasing CH_4_ emissions is especially high for alder stands that are directly connected with constant water bodies such as rivers and lakes as is common in northern and central Europe (Claessens et al., 2010).

## 5 CONCLUSIONS

The importance of stem CH_4_ emissions may vary considerably between individual ecosystems, even if dominated by the same tree species. Here, we show that the variability of stem and soil CH_4_ emissions cannot be explained by a single influential factor. Further, not all dry forests necessarily show stem CH_4_ emissions and emissions from wetland trees may vary considerably in the short‐term and between years. Although our findings suggest the soil to be the origin of the CH_4_ coming from the stems, the observed variability of this emission path and the unknown transport mechanism inside the tree clearly ask for more mechanistic research within this topic. Furthermore, to account for the variability between ecosystems, constantly wet alder forests that are spatially important in central Europe need also to be investigated thoroughly in the future.

## CONFLICTS OF INTEREST

The authors declare no conflict of interest.

## AUTHOR CONTRIBUTIONS

D.K., A.G., and G.J. contributed to design of the research. D.K. and I.S. contributed to field work. D.K., A.G., I.S., and G.J. contributed to data analysis, collection, or interpretation. D.K., A.G., and G.J. involved in writing the manuscript.

## FUNDING INFORMATION

The European Social Fund (ESF) and the Ministry of Education, Science and Culture of Mecklenburg‐Western Pomerania (Germany) funded this work within the scope of the project WETSCAPES (ESF/14‐BM‐A55‐0030/16).

## Supporting information

Fig S1

Fig S2

Fig S3

Table S1

Table S2

Table S3

## Data Availability

Data is accessible via PANGAEA link:https://doi.pangaea.de/10.1594/PANGAEA.922558
