# Supplementary figures and images for: Short‐lived peaks of stem methane emissions from mature black alder (Alnus glutinosa (L.) Gaertn.) – Irrelevant for ecosystem methane budgets?
Source: Plant Environ Interact. 2020 Dec 23;2(1):16–27. doi: 10.1002/pei3.10037 (PMC10168070; doi:10.1002/pei3.10037)

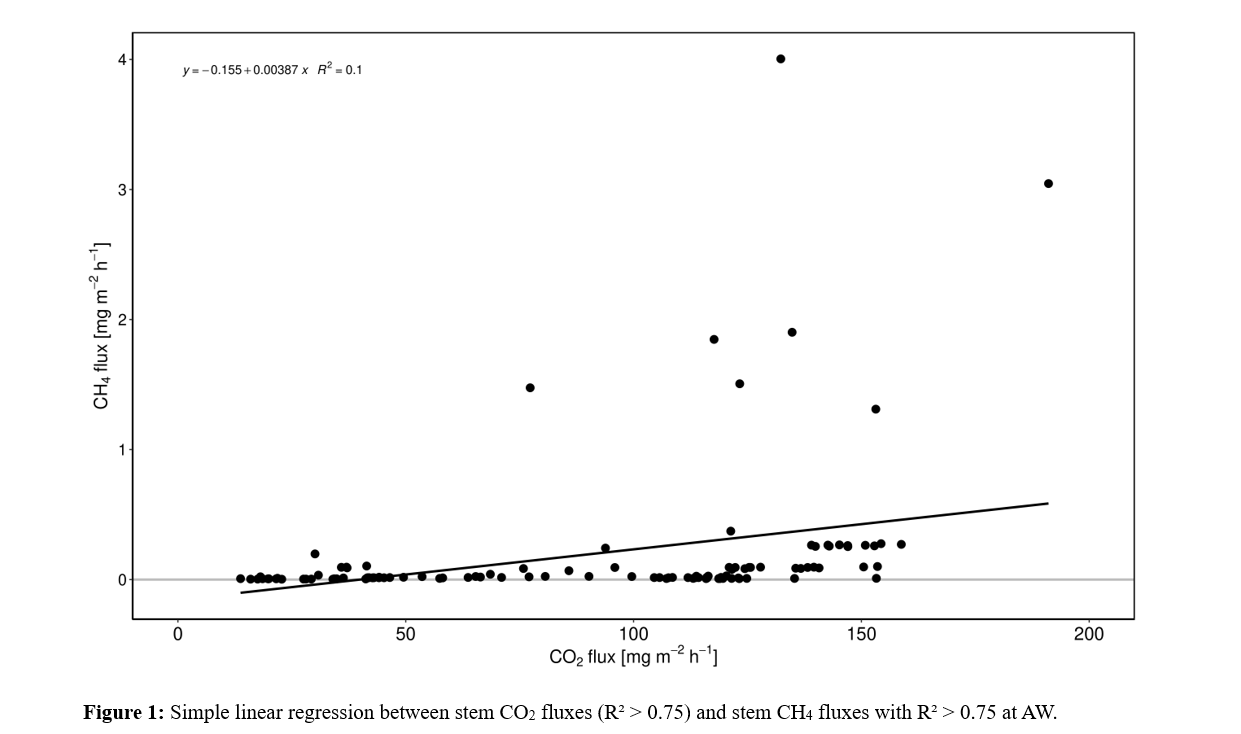

Supplement: Supplementary file 1 — Fig S1 [file PEI3-2-16-s005.png]

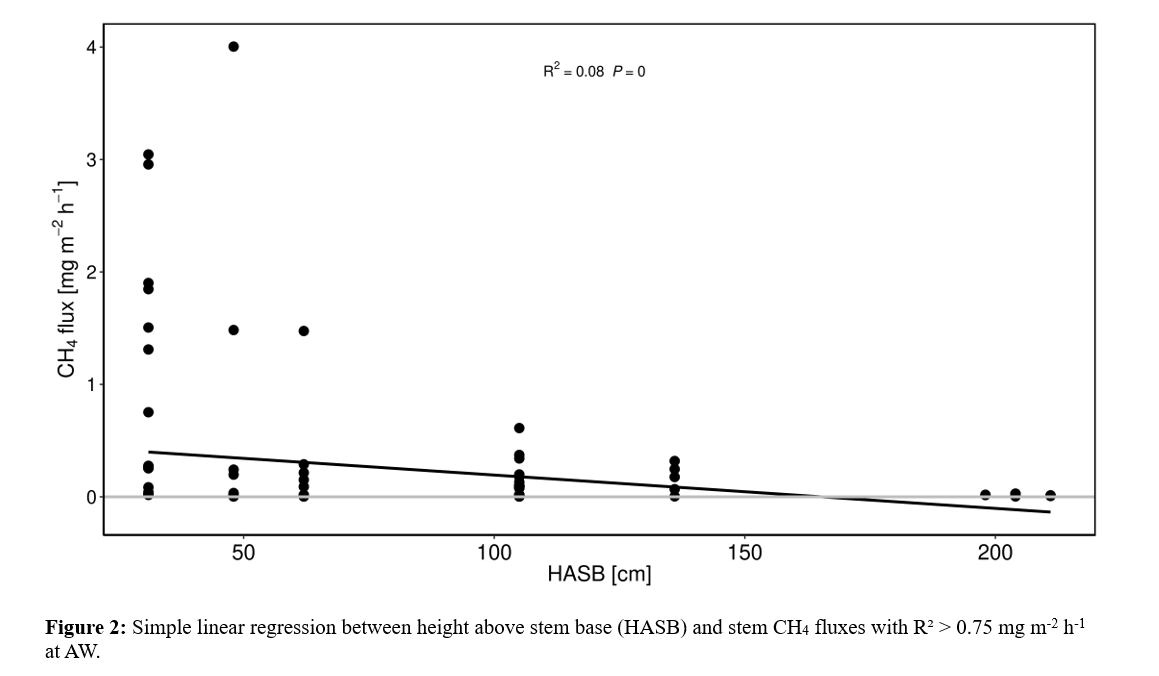

Supplement: Supplementary file 2 — Fig S2 [file PEI3-2-16-s004.png]

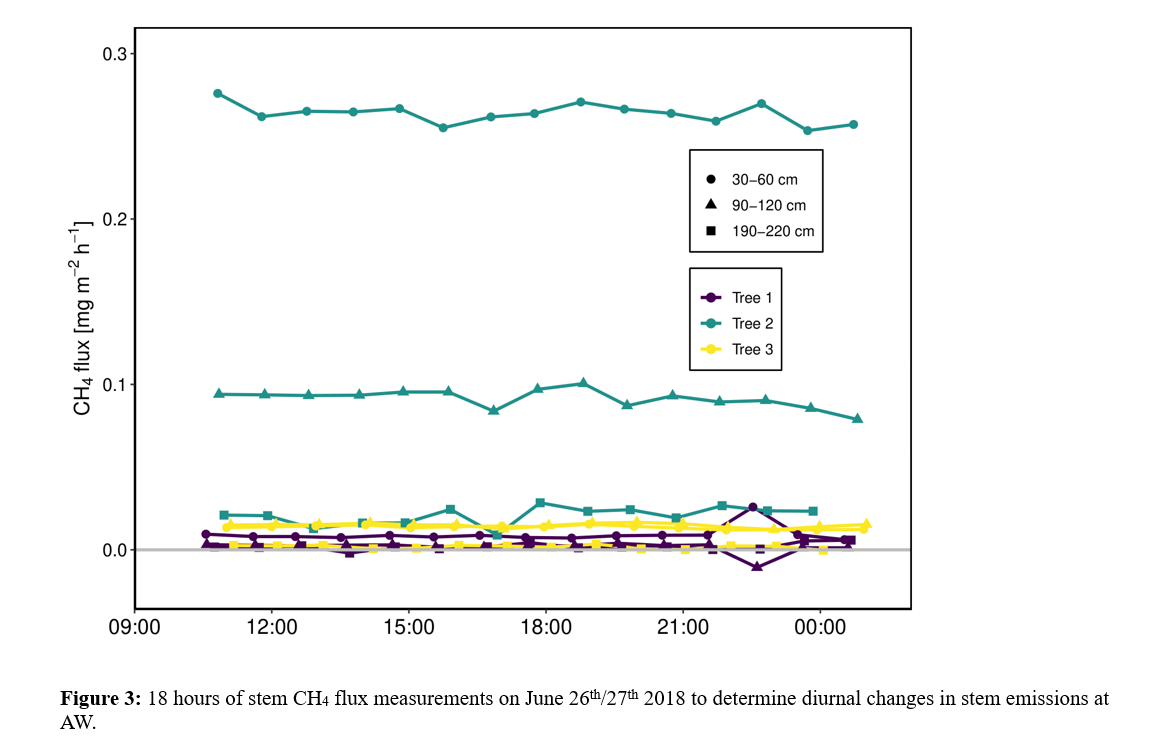

Supplement: Supplementary file 3 — Fig S3 [file PEI3-2-16-s003.png]
