# Supplementary material for: Short‐lived peaks of stem methane emissions from mature black alder (Alnus glutinosa (L.) Gaertn.) – Irrelevant for ecosystem methane budgets?
Source: Plant Environ Interact. 2020 Dec 23;2(1):16–27. doi: 10.1002/pei3.10037 (PMC10168070; doi:10.1002/pei3.10037)
Supplement: Supplementary file 4 — Table S1 [file PEI3-2-16-s006.docx]

**Table S1:** Model summary for linear regression between stem CO_2_ flux and stem CH_4_ flux at AW.

| Model | R² | Adjusted R² | F statistic | Degrees of freedom | p-value |
| --- | --- | --- | --- | --- | --- |
| CH_4_ vs. CO_2_ | 0.1 | 0.09 | 12.3 | 110 | <0.01 |
|  |  |  |  |  |  |
