# Supplementary material for: Short‐lived peaks of stem methane emissions from mature black alder (Alnus glutinosa (L.) Gaertn.) – Irrelevant for ecosystem methane budgets?
Source: Plant Environ Interact. 2020 Dec 23;2(1):16–27. doi: 10.1002/pei3.10037 (PMC10168070; doi:10.1002/pei3.10037)
Supplement: Supplementary file 5 — Table S2 [file PEI3-2-16-s002.docx]

**Table 2:** Model summary for exponential linear regression between stem height above stem base (HASB) and stem CH_4_ fluxes with R² > 0.75 at AW.

| Model | R² | Adjusted R² | F statistic | Degrees of freedom | p-value |
| --- | --- | --- | --- | --- | --- |
| log(CH_4_) vs. HASB | 0.08 | 0.07 | 11.37 | 137 | <0.01 |
|  |  |  |  |  |  |
