# Supplementary material for: Short‐lived peaks of stem methane emissions from mature black alder (Alnus glutinosa (L.) Gaertn.) – Irrelevant for ecosystem methane budgets?
Source: Plant Environ Interact. 2020 Dec 23;2(1):16–27. doi: 10.1002/pei3.10037 (PMC10168070; doi:10.1002/pei3.10037)
Supplement: Supplementary file 6 — Table S3 [file PEI3-2-16-s001.docx]

**Table 3:** Summary statistics of linear and exponential linear regression models of stem fluxes (AWT) and soil fluxes AW) with daily mean air temperature [°C] and daily mean water level [cm].

| AWT |  |  |  |  |  |  |
| --- | --- | --- | --- | --- | --- | --- |
| Model | Type | R² | Adjusted R² | F statistic | Degrees of freedom | p-value |
| Air temperature | exponential | 0.239 | 0.237 | 110.6 | 352 | <0.01 |
| Air temperature | linear | 0.015 | 0.012 | 5.474 | 352 | 0.02 |
| Water level | exponential | 0.228 | 0.226 | 104.1 | 352 | <0.01 |
| Water level | linear | 0.051 | 0.047 | 18.77 | 352 | <0.01 |
| Soil temperature | exponential | 0.234 | 0.231 | 107.4 | 352 | <0.01 |
| Soil temperature | linear | 0.016 | 0.013 | 5665 | 352 | 0.02 |
|  |  |  |  |  |  |  |
| AW |  |  |  |  |  |  |
| Model | Type | R² | Adjusted R² | F statistic | Degrees of freedom | p-value |
| Air temperature | exponential | 0.210 | 0.201 | 18.87 | 70 | <0.01 |
| Air temperature | linear | 0.094 | 0.087 | 7302 | 70 | 0.012 |
| Water level | exponential | 0.079 | 0.066 | 5987 | 70 | 0.017 |
| Water level | linear | 0.005 | 0.005 | 0.3866 | 70 | 0.598 |
| Soil temperature | exponential | 0.261 | 0.251 | 24.75 | 70 | <0.01 |
| Soil temperature | linear | 0.154 | 0.141 | 27364 | 70 | <0.01 |
